# Effect of swab pooling on the Accula point-of-care RT-PCR for SARS-CoV-2 detection

**DOI:** 10.3389/fmicb.2023.1219214

**Published:** 2023-08-07

**Authors:** Moira Lancelot, Kirby Fibben, Julie Sullivan, William O’Sick, Kaleb McLendon, Huixia Wu, Anuradha Rao, Leda C. Bassit, Morgan Greenleaf, Pamela Miller, Wolfgang Krull, Erika Tyburski, John D. Roback, Wilbur A. Lam, Gregory L. Damhorst

**Affiliations:** ^1^Department of Pathology and Laboratory Medicine, Emory University School of Medicine, Atlanta, GA, United States; ^2^Wallace H. Coulter Department of Biomedical Engineering, Georgia Institute of Technology, Atlanta, GA, United States; ^3^The Atlanta Center for Microsystems-Engineered Point-of-Care Technologies, Atlanta, GA, United States; ^4^Department of Pediatrics, Emory University School of Medicine and Children’s Healthcare of Atlanta, Atlanta, GA, United States; ^5^Laboratory of Biochemical Pharmacology, Emory University, Atlanta, GA, United States; ^6^Rapid Acceleration of Diagnostics (RADx), Maryland, MD, United States; ^7^Aflac Cancer & Blood Disorders Center at Children's Healthcare of Atlanta, Atlanta, GA, United States; ^8^Division of Infectious Diseases, Department of Medicine, Emory University School of Medicine, Atlanta, GA, United States

**Keywords:** SARS-CoV-2, RT-PCR, swab pooling, screening, point-of-care

## Abstract

**Introduction:**

Swab pooling may allow for more efficient use of point-of-care assays for SARS-CoV-2 detection in settings where widespread testing is warranted, but the effects of pooling on assay performance are not well described.

**Methods:**

We tested the Thermo-Fisher Accula rapid point-of-care RT-PCR platform with contrived pooled nasal swab specimens.

**Results:**

We observed a higher limit of detection of 3,750 copies/swab in pooled specimens compared to 2,250 copies/swab in individual specimens. Assay performance appeared worse in a specimen with visible nasal mucous and debris, although performance was improved when using a standard laboratory mechanical pipette compared to the transfer pipette included in the assay kit.

**Conclusion:**

Clinicians and public health officials overseeing mass testing efforts must understand limitations and benefits of swab or sample pooling, including reduced assay performance from pooled specimens. We conclude that the Accula RT-PCR platform remains an attractive candidate assay for pooling strategies owing to the superior analytical sensitivity compared to most home use and point-of-care tests despite the inhibitory effects of pooled specimens we characterized.

## 1. Introduction

The COVID-19 pandemic has resulted in more than 700 million confirmed cases and nearly 7 million deaths worldwide (WHO, 2023). This global public health emergency underscored the need for frequent, affordable, and accessible testing for SARS-CoV-2. Despite the vaccination of more than 5 billion individuals worldwide (WHO, 2023), SARS-CoV-2 infection remains a threat to many individuals, particularly those with medical comorbidities and immune compromise (Hu et al., 2023; Malahe et al., 2023). Tremendous effort by academic and industry players has accelerated the development, manufacturing, validation, and dissemination of new diagnostic assays – particularly those designed for implementation at the point-of-care (PoC) or by consumers in the home (Tromberg et al., 2020).

**Figure 1:**
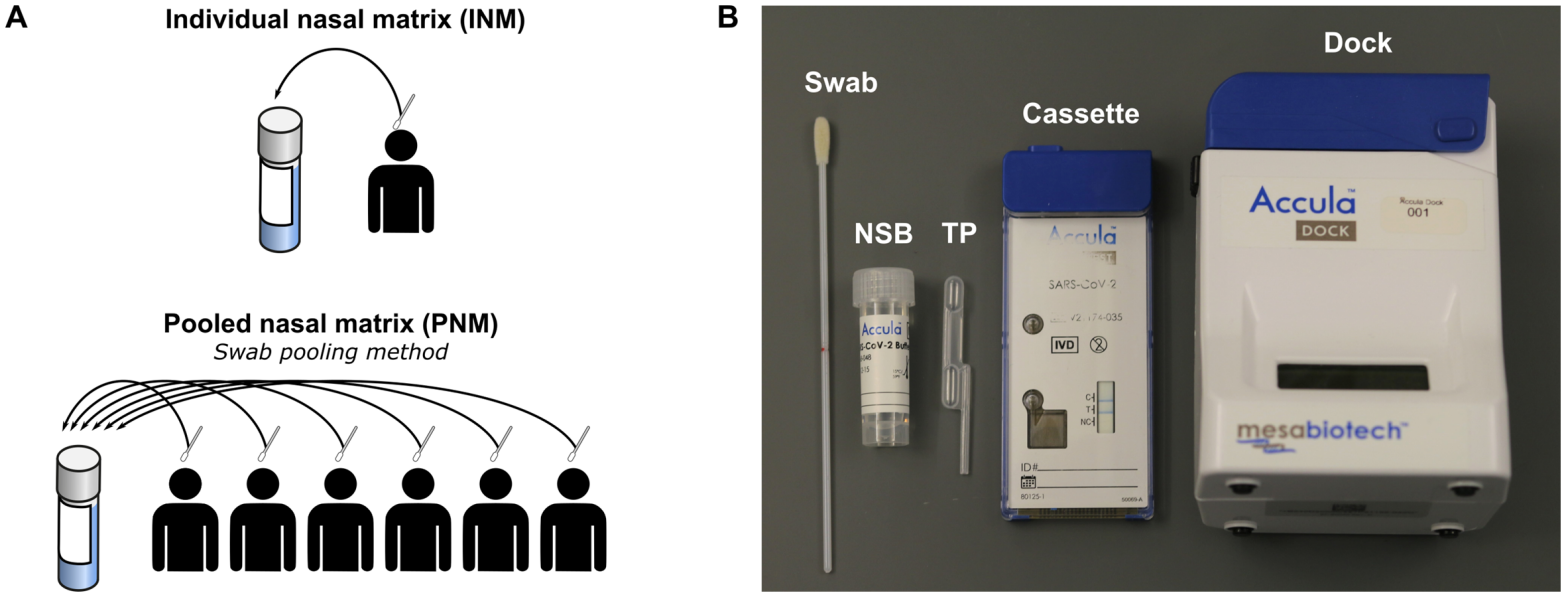
**(A)** Schematic of negative matrix preparation. **(B)** Components of Accula assay and kit. NSB, nasal swab buffer; TP, transfer pipette; INM, individual nasal matrix; PNM, pooled nasal matrix.

The availability of new diagnostic tools has enabled public health efforts involving novel testing strategies that maximize limited resources and optimize public safety. Pooling allows for testing large groups of people while minimizing the consumption of limited resources, and is appropriate for settings where prevalence of the infection is sufficiently low that most individuals will test negative (Lagopati et al., 2021; Millioni and Mortarino, 2021). Pooling strategies employ either swab pooling or sample pooling to perform a single test for a group of individuals. Swab pooling, the method used in this study, involves eluting swabs from multiple patients into a single volume of transport media (Figure 1), (RADx, 2022). Sample pooling (or media pooling) involves combining aliquots of transport media collected separately from multiple individuals (Supplementary Figure S1) (RADx, 2022). With either method, a positive result would necessitate re-testing everyone in the pool, but a negative result indicates that all samples in the pool were negative. Any assay employed in a pooling strategy must exhibit sufficient sensitivity in the analysis of pooled specimens, and validation under these conditions is warranted. With generally superior performance to rapid antigen tests, PoC assays based on nucleic acid amplification are the ideal candidates for a pooling-based testing program, although most are not approved for use outside of a controlled environment.

The Thermo-Fisher Accula is a rapid PoC platform for RT-PCR that has been FDA-approved for detecting influenza A and B, respiratory syncytial virus, and SARS-CoV-2 (Hogan et al., 2020; Greenleaf et al., 2022; USFDA, 2022). The Accula SARS-CoV-2 test has been assigned a certificate of laboratory improvements amendments (CLIA) waiver (Greenleaf et al., 2022; USFDA, 2022), and its small form factor and ease of use could enable testing in near-patient and mobile settings such as schools, workplaces, and events when operated by skilled users in a certified, controlled environment.

Testing pooled samples may introduce challenges not encountered with individual testing. Chief among these is the potential for compounding interfering substances in the pooling scenario. Consider nasopharyngeal sampling, where a swab is inserted into the nares or nasopharynx and then eluted into a liquid buffer. When a pooling strategy involves eluting swabs obtained from multiple individuals into a shared buffer, nasal mucous, inhaled medications, and other contaminants would be contributed from everyone in the pool, which may impact assay performance through additive effects. Further, a single inhibitor introduced from just one member of the pool would compromise the entire pool and, particularly if the result is falsely negative, undermine the pooling strategy.

In this report, we describe the performance of the Thermo-Fisher Accula RT-PCR for contrived pools of six nasal swab specimens. We observe a reduction in assay performance which we postulate is mainly due to the presence of nasal mucous and debris. Although this appears to reduce the assay’s sensitivity, it is still an attractive candidate for PoC pooling strategies owing to the superior sensitivity of RT-PCR compared to antigen tests.

## 2. Materials and methods

### 2.1. Heat-inactivated SARS-CoV-2

All Accula experiments were performed with the following reagent deposited by the Centers for Disease Control and Prevention and obtained through BEI Resources, NIAID, NIH: Quantitative PCR (qPCR) Extraction Control from Heat-Inactivated SARS-Related Coronavirus 2, Isolate United States-WA1/2020, NR-52350, lot 70036662.

### 2.2. Nasal swab

The FLOQSwabs® 502CS01 Regular Size Nylon® Flocked Swab (Copan Diagnostics, CA, United States) was used for experiments and collection of Negative Nasal Matrix.

### 2.3. Negative matrix specimens

Negative matrix specimens were prepared from volunteers recruited at Thermo Fisher, Emory University, or community testing sites in the metro Atlanta area following provision of informed consent under protocols approved by the institutional review boards of Mesa/Thermo Fisher or Emory University (STUDY00001082) and in accordance with relevant guidelines and regulations. Active recruitment at Emory University and metro Atlanta testing sites occurred between July 6, 2020 and October 12, 2021. The authors did not have access to identifying information during or after experiments. Volunteers contributing to the negative matrix performed self-swabbing of the anterior nares from both nostrils according to the Accula instructions for use (IFU) (Mesabiotech, 2021). Pooled nasal matrix (PNM) was prepared with swabs from six volunteers eluted into a common nasal swab buffer (NSB; Figure 1A) provided in the Accula test kit using the technique described in the IFU (Mesabiotech, 2021). Each swab was removed and discarded before the next swab was inserted. After assembly of a complete pool of six swabs, a single Accula assay was performed to verify negativity and the remaining specimen was frozen at −80°C for storage until experiments were performed. NSB has a starting volume of 5 mL, but the true final volume of the specimen was approximately 4–4.5 mL owing to loss of volume during swab elution and the initial screening assay. Individual nasal matrix (INM) was also prepared according to the Accula IFU with elution of a single swab into a single NSB vial subsequently frozen at −80°C. Each INM was tested with a single Accula assay either before or after freezing to confirm negativity.

### 2.4. Specimen spiking with heat-inactivated virus

PNM and INM limit of detection (LOD) studies followed a protocol similar to those used in recent US Food and Drug Administration (FDA) Emergency Use Authorization filings in which 50 μL of diluted NR-52350 was placed on a dry swab prior to introducing the swab into the PNM or INM (Detect, 2021; USFDA, 2021). An alternate method was used to spike the near-LOD experiments, where target concentrations of 100 genome copies per mL or 300 genome copies per mL were selected because a LOD of 150 copies per mL was described in initial FDA Emergency Use Authorization (EUA) documents for the Accula assay. In the near-LOD experiments, NR-52350 was spiked directly into PNM by performing serial dilutions in NSB as needed and pipetting a small volume of diluted NR-52350 into PNM to achieve the intended concentration. As a result, target copy numbers described are not directly comparable between the LOD-determining and near-LOD experiments.

### 2.5. Accula RT-PCR assay

The Accula RT-PCR assay consists of a single-use cassette and a multiple-use reader (Figure 1B). All assays were performed according to the manufacturer’s IFU except for those in which a mechanical pipette (MP) was used to load the sample into the cassette in place of the fixed-volume Transfer Pipette (TP) provided with the Accula kit (Mesabiotech, 2021). Results of the assay are indicated by presence or absence of a blue line in a window on the cartridge.^4^ Results for all experiments were observed within 15 min of assay completion.

### 2.6. Determination of LOD with a spiked swab

Two dry swabs were spiked with 50 μL of NSB containing 250, 750, 1,500, 2,250, 3,000, 3,750, or 4,500 copies of NR-52350. One swab for each copy number was then eluted into one PNM, and its pair was eluted into one INM specimen and subsequently stored on ice. Three assays were performed on each specimen. The lowest copy number specimen with 3 of 3 positive results was then used to perform 20 additional assays. If fewer than 19 of these assays were positive, 20 assays were performed from the next highest copy number specimen. This was repeated until at least 19 of 20 assays were positive.

### 2.7. Specimens for swab sequence testing

For swab sequence testing, six asymptomatic volunteers were provided with three swabs each. To minimize the potential confounding by repetitive swabbing, collection was performed in the following sequence: Swab A was inserted into the left nostril and the circumference of the anterior nares was brushed 10 times in accordance with the Accula IFU. Next, swab B was inserted into the right nostril with brushing. Then, the right nostril was brushed with swab A followed by brushing of the left nostril with swab B. Finally, swab C was collected by brushing both nostrils without specification of which nostril should be swabbed first.

Swab C from each of the six volunteers was used to perform an individual test to verify the volunteer was negative for SARS-CoV-2. Two pools were created from swabs A and B from the six volunteers (Table 1). Pool I (“first in” pool) was created by spiking swab A from volunteer 1 with 50 μL NSB containing 4,000 genome copies NR-52350 and eluting it into NSB followed by swab A from volunteer 2, swab A from volunteer 3, and swab B from volunteers 4, 5 and 6 in sequence. Similarly, pool II (“last in” pool) was created by eluting swab B from volunteers 1, 2 and 3 sequentially, followed by swab A from volunteers 4 and 5. Swab A from volunteer 6 was the final swab to be added to pool II and was spiked with 50 μL containing 4,000 genome copies NR-52350 prior to elution.

**Table 1:** Sequence of swab addition to create positive pools for swab sequence testing.

	Sequence	Pool I (“first in”)	Pool II (“last in”)
Volunteer A	1	Swab A, spiked with NR-52350	Swab B
Volunteer B	2	Swab A	Swab B
Volunteer C	3	Swab A	Swab B
Volunteer D	4	Swab B	Swab A
Volunteer E	5	Swab B	Swab A
Volunteer F	6	Swab B	Swab A, spiked with NR-52350

A third swab, Swab C, was used to individually test each volunteer to confirm the absence of natural SARS-CoV-2 in the contributed specimens.

### 2.8. Limit of detection comparisons

To aid in the interpretation of the LOD determined by pooling experiments with the Accula in the context of other PoC and home-use tests, we referenced data generated at our institution from LOD experiments performed with several common SARS-CoV-2 antigen assays and the LOD claimed in FDA EUA submission data for CLIA-waived PoC molecular assays.

The LOD experiments for antigen tests were performed by preparing a panel of dilutions using pooled remnant clinical samples with SARS-CoV-2 lineage B.1.2 in nasal wash or nasal swab matrix according to the matrix most compatible with each assay. Rao et al. (2022) an aliquot of 50 μL of each dilution was added to the swab used with each assay kit, and the test was performed according to the IFU for that test. This was repeated for a total of 3 replicates. LOD was determined from the highest dilution (lowest RNA copy number) where at least 2 of 3 replicate assays were positive. To determine the RNA copy number of the highest dilution producing 2 of 3 positive tests, RNA was isolated from 140 μL of the diluted sample in the matrix, and RT-PCR for the N2 gene was performed using 2019-nCoV CDC EUA Kit, 1,000 reaction combined Primer/Probe Mix (N2 gene) (IDT, Catalog No. 10006770) in a LightCycler 480 II instrument (Roche). A quantitative synthetic RNA from SARS—Related Coronavirus 2 (BEI, cat# NR-52358; lot# 70035241, stock at 1.05×10^8^ genome equivalents/mL) was used simultaneously as a calibration from which RNA copy number per mL was calculated. A standard curve for the N2 gene primers was generated by diluting the synthetic RNA 10-fold and amplified by quantitative RT-PCR reaction.

The claimed LOD for molecular tests was determined by referencing the IFU published on the FDA website. USFDA, (2021) only those assays which report LOD in terms of viral genome copies per swab were considered. Some EUA submissions report viral genome copies per milliliter (or microliter) of buffer or transport media, which is not directly comparable to the LOD measurements performed with the Accula system in this study. Assays with a format incompatible with swab pooling are not included in the comparison.

## 3. Results

### 3.1. Higher LOD in PNM specimens

**Figure 2:**
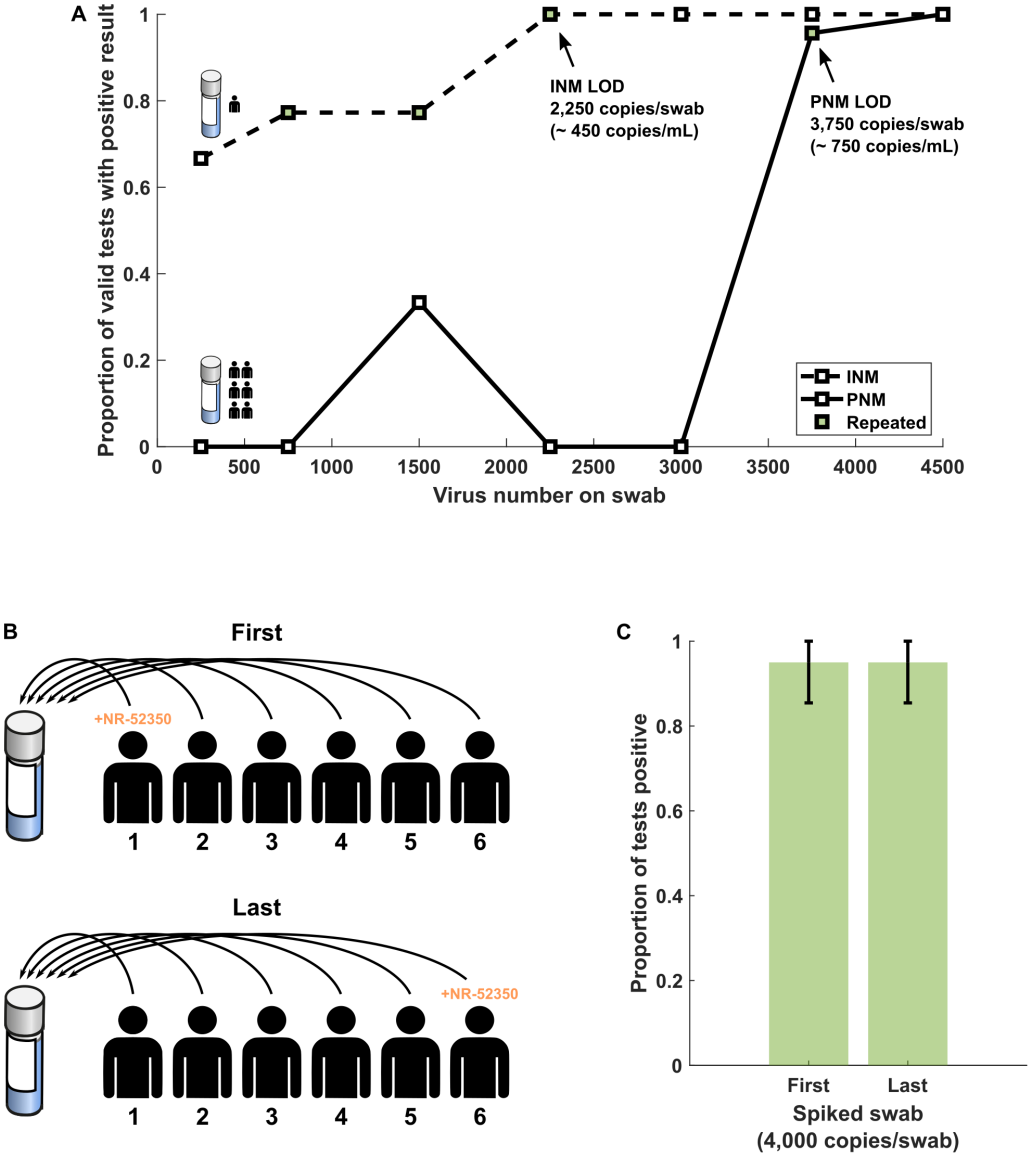
**(A)** Results of range-finding studies using INM and PNM suggest that the LOD is higher in PNM samples. **(B)** Schematic of swab sequence experiment protocol in which the first or last swab from a sequence of six SARS-CoV-2 negative volunteers was spiked with NR-52350. **(C)** At a target copy number near the LOD for PNM (4,000 copies per swab), the performance of the assay is not significantly different regardless of when in the pooling sequence the positive swab is introduced. Error bars represent the 95% confidence interval using the formula for standard error. INM, individual nasal matrix; PNM, pooled nasal matrix; LOD, limit of detection.

Initial assays produced 3 of 3 positive results from INM specimens eluted with 750 copies and greater. Twenty-replicate reflex testing of identical specimens produced 14 positive, 5 negative, and 1 invalid result from both 750 and 1,500 copy specimens (Table 2, Figure 2A). The 2,250 copy number specimen produced 19 positive and 1 invalid result. Initial assays produced 3 of 3 positive results from PNM specimens with 3,750 and 4,500 copies. Reflex testing of the 3,750-copy specimen produced 19 positive and 1 negative result (Figure 2A). These data convey an LOD based on the spiked-swab protocol for the Accula RT-PCR assay of 2,250 copies (approximately 450 copies/mL buffer) in INM and 3,750 copies (approximately 750 copies/mL buffer) in PNM.

**Table 2:** Results of limit of detection experiments demonstrated LOD for INM of 2,250 RNA copies/swab and for PNM of 3,750 RNA copies/swab.

RNA copies/swab	INM					PNM				
	Initial		Repeat			Initial		Repeat		
	+	–	+	–	I	+	–	+	–	I
250	2	1	··	··	··	0	3	··	··	··
750	3	0	14	5	1	0	3	··	··	··
1,500	3	0	14	5	1	1	2	··	··	··
2,250	3	0	19	0	1	0	3	··	··	··
3,000	3	0	··	··	··	0	3	··	··	··
3,750	3	0	··	··	··	3	0	19	1	0
4,500	3	0	··	··	··	3	0	··	··	··

INM, individual nasal matrix; PNM, pooled nasal matrix; I, invalid.

### 3.2. Swab sequence does not significantly affect assay performance

Twenty assays were performed from two newly prepared, never-frozen PNM from six volunteers. The PNM specimen prepared by spiking the first of six swabs produced 19 positive and 1 invalid result (Figure 2C). The PNM specimen prepared by spiking the last of six swabs produced 19 positive and 1 negative result (Figure 2C).

### 3.3. Near-LOD assay performance is inhibited in the presence of visible debris

**Figure 3:**
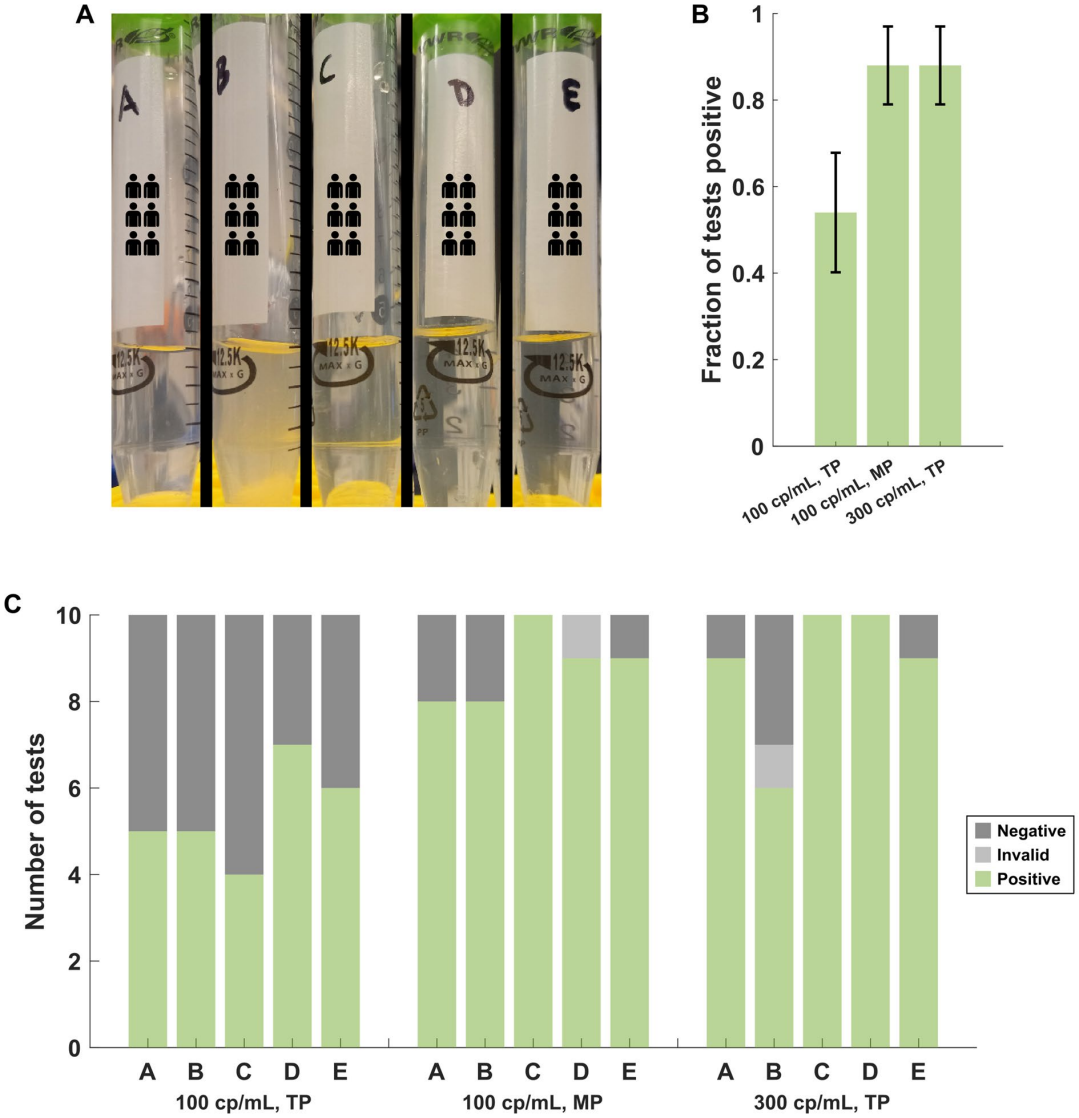
**(A)** Appearance of the PNM specimens used in the near-LOD experiments comparing pipettor and target copy number. Specimen B was more turbid, and debris was observed. **(B)** Cumulative performance of the 5 samples under each target copy number and pipettor condition demonstrates poor performance at low copy numbers with the TP but improved performance with the MP at the same copy number or with a higher target copy number while using the TP. Error bars represent the 95% confidence interval using the formula for standard error. **(C)** The same results as (B), separated by the specimen, demonstrate that specimen B produced poor results with the TP at the higher copy number that was incongruent with the results from the other 4 specimens. TP, transfer pipette; MP, mechanical pipette.

Five PNM specimens (A-E) were spiked with heat-inactivated SARS-CoV-2 (BEI cat# NR-52350: United States-WA1/2020) to an approximate concentration of 100 genome copies per mL. Specimen B was noted to be more turbid than the other four specimens with visible debris (Figure 3A). Ten assays for each specimen were performed with the transfer pipette (TP) followed by ten assays from each specimen with the MP. Additional NR-52350 was then added to each specimen to achieve a concentration of 300 genome copies per mL, and ten assays were performed from each specimen with the TP. Cumulative results demonstrated poor performance (27/50 positive tests) at the low concentration (100 cp/mL) when the TP was used for specimen loading (Figure 3B). This was overcome by using the MP for sample loading with the low concentration (44/50 positive tests) or by testing a higher concentration specimen (300 cp/mL) with the TP (44/50 positive tests). Examination of the same results individually within each specimen suggests that the turbid sample still performed poorly at higher target concentrations (6/10 positive tests; Figure 3C).

### 3.4. Near-LOD assay inhibition is not due to transfer pipette leeching or binding

**Figure 4:**
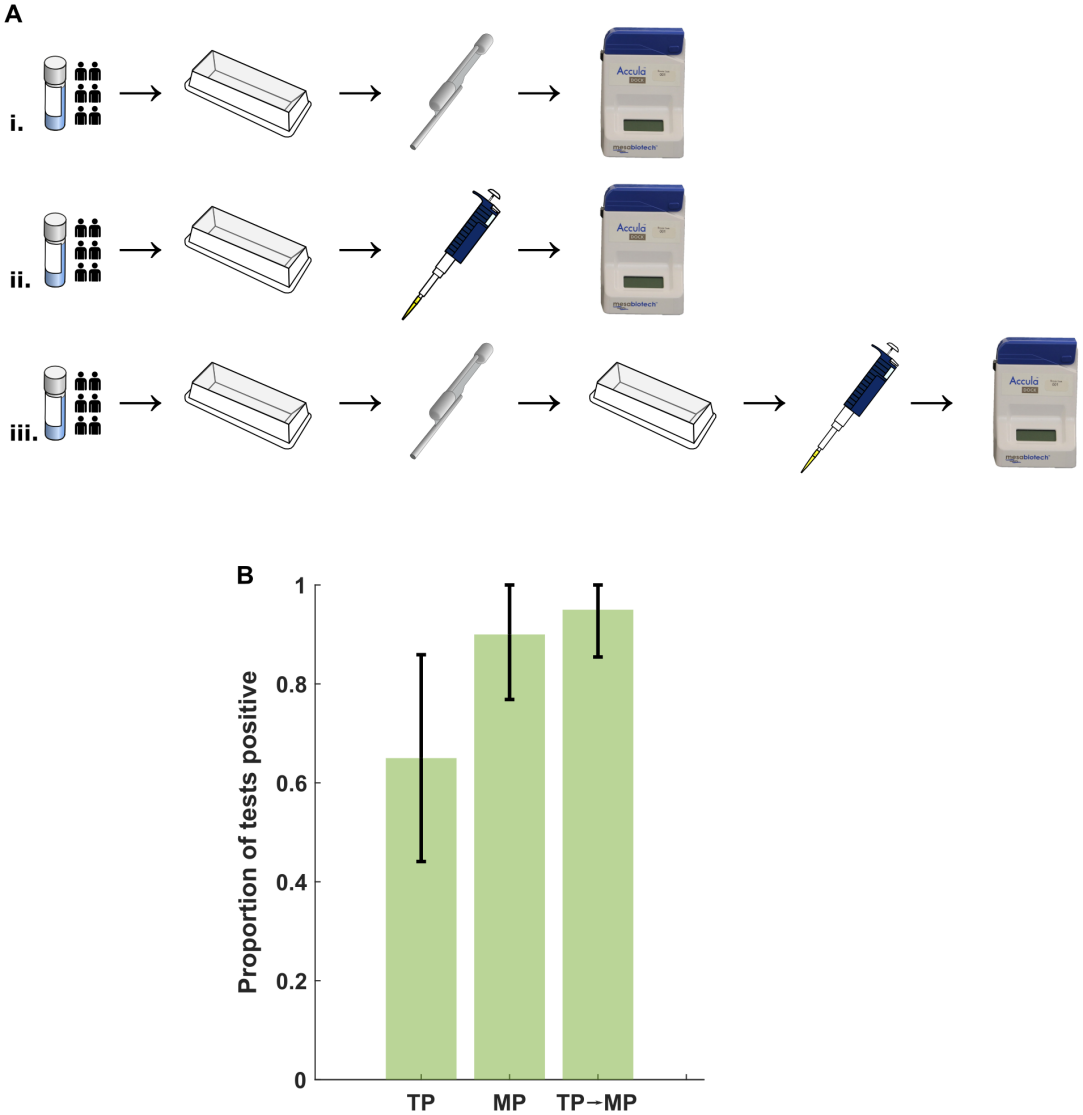
**(A)** Protocol for examination of deleterious effects from the TP. Specimen was prepared in a reagent tray and (i) transferred to the Accula cassette using the TP, (ii) transferred to the cassette using an MP, or (iii) transferred to a secondary container with the TP followed by transfer to the cassette with an MP. TP, transfer pipette; MP, mechanical pipette. **(B)** No significant difference in the proportion of positive tests after transfer with the TP and assay loading with the MP. Error bars represent the 95% confidence interval using the formula for standard error. TP, transfer pipette; MP, mechanical pipette.

To examine potential deleterious effects of the TP independent of nasal mucous and debris, NR-52350 was diluted in NSB to approximately 100 cp/mL concentration. 20 assays were performed under the following conditions: (i) TP specimen loading, (ii) MP specimen loading and (iii) transfer of specimen into a secondary container with the TP followed by loading of the transferred sample into the assay cassette with the MP (Figure 4A). Results summarized in Figure 4B demonstrate impaired assay performance with TP loading (13/20 positive tests) that was not reproduced in condition 3 with TP transfer followed by MP loading (19/20 positive tests), suggesting the TP may introduce an additional inhibitory factor other than leeching such as air bubbles that interfere with the microfluidic system.

### 3.5. Limit of detection comparisons

**Figure 5:**
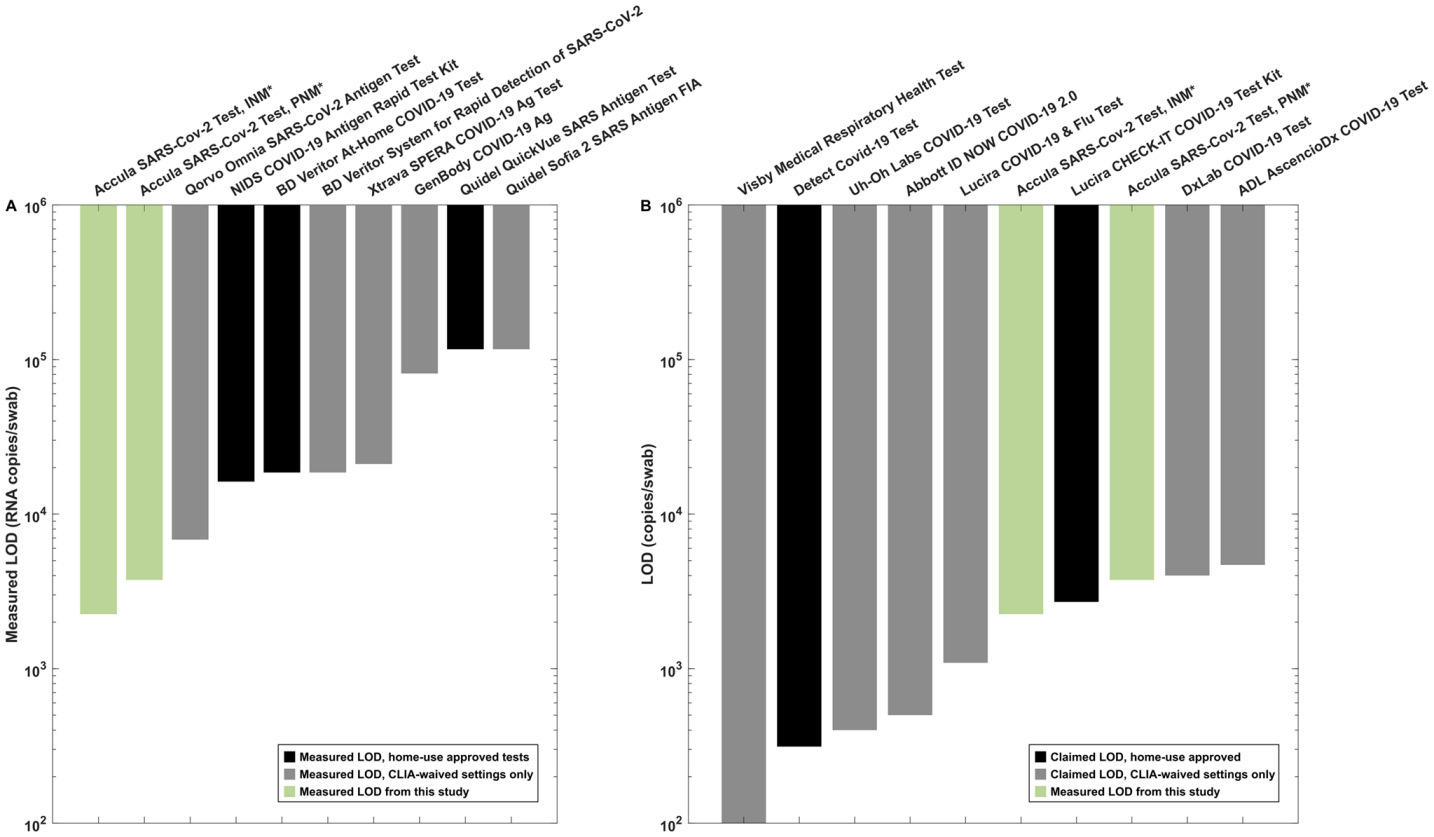
Reference data for interpretation of the measured LOD in INM and PNM. **(A)** Antigen test LOD measurements from our institution using SARS-CoV-2 lineage B.1.2. All experiments other than the Accula experiments were performed in matrix-simulating specimens derived from a single individual (i.e., INM) as part of a separate testing program at our institution. **(B)** Claimed LOD for CLIA-waived molecular assays (black bars) as listed in IFU documents on the FDA website compared to measured INM and PNM LOD for the Accula (green bars). *Indicates the experiments performed for pooling testing as presented in detail in this manuscript.

LOD testing experiments from 8 antigen-based SARS-CoV-2 assays performed using individual nasal matrix compatible with each test suggested that none of the common assays exhibited a lower LOD than the Accula platform with PNM (Figure 5A and Supplementary Table S1). The measured LOD for the Abbott BinaxNOW COVID-19 Ag (948 copies/swab) is not displayed in the figure because the assay format is incompatible with swab pooling.

We also compared our results to the claimed LOD for molecular tests with EUA as listed on the FDA website (Figure 5B). These assays generally exhibited similar or lower LOD for individual specimen testing compared to the PNM LOD measured in our experiments using the Accula system.

## 4. Discussion

Strategies for promoting the safe gathering of groups of people in workplaces, schools and at large events has varied across the course of the COVID-19 pandemic. While individuals with symptoms of illness should always be encouraged to avoid others, the potential to spread SARS-CoV-2 exists in individuals with subclinical or absent symptoms. To minimize risk, some programs and institutions have required proof of vaccination or even employed active testing programs. When a testing strategy is desired, various approaches can be considered. Individual testing with home use diagnostic tests may be prohibitively costly and produce significant waste, and many home use tests suffer from inferior sensitivity. The swab pooling strategy promises more efficient use of testing resources while allowing for use of more sophisticated (and likely more sensitive) diagnostic instruments, yet increased test turnaround time and the added expense of transporting specimens may prohibit swab pooling in a centralized laboratory.

Swab pooling with a PoC instrument may uniquely meet the needs of many events or institutions by allowing pooled testing in the proximity of a large gathering. However, the impact of any pooling strategy for PoC SARS-CoV-2 diagnostics has only been described in a few studies (Becker et al., 2020; Burdett et al., 2021; Heaney et al., 2022; Lee et al., 2022; Yang et al., 2022) and to our knowledge has not previously been investigated using the Accula platform. Our experiments with contrived specimens on the Accula SARS-CoV-2 RT-PCR assay reveal a higher LOD when a pooled specimen is compared to an individual specimen. Still, assay performance did not appear to be affected by the position of the positive swab in the pooling sequence.

We hypothesized that impaired assay performance of pooled specimens could be attributed to an overall greater burden of nasal mucous and debris as swabs from multiple individuals are eluted into the same volume of buffer designed for individual testing. The observation of fewer false negative and invalid results when loading PNM specimens at 100 copies/mL (slightly below the LOD reported in the Accula EUA) with an MP compared to a TP has led us to postulate that the smaller bore of the MP may filter some particulate or produce shear forces that achieve greater sample homogenization and reduce the burden of these inhibitors in the specimen. TP-loaded specimens with 300 copies/mL appeared to overcome this problem, suggesting this inhibition is not as significant at higher target concentrations. We observed that the sample with distinctly greater turbidity and particulate contents uniquely performed worse despite the higher target concentration, suggesting that the deleterious effect of nasal mucous and debris likely also depends on the amount of the nasal mucous. These observations indicate that pooling strategies for PoC assays must consider the compounding of nasal mucous and debris. For example, anticipatory guidance in pooling IFU could warn the assay operator that visibly turbid pooled specimens may warrant re-testing of individuals separately to avoid the undesirable effects of a single individual who contributes high quantities of mucous.

Our experiments in buffer with intermediate transfer of specimen using the TP rule out a component of leeching chemical inhibitor from the TP plastic material or binding of viruses or viral RNA to the TP. However, the observation that the TP-loaded specimens performed worse than the MP-loaded specimens (with or without intermediate transfer using the TP) suggests that there is also a disruptive effect from the TP independent of the presence of nasal mucous. We speculated that this might be due to the TP’s introduction of air bubbles to the microfluidic cartridge, which could inhibit the assay. This phenomenon warrants further investigation to weigh the negative effects of the TP on assay performance against the need for a single-use, fixed-volume tool that may not provide the precision of a standard laboratory micropipette.

Our observations of the effects of pooling on the performance of the Accula assay are limited by use of contrived specimens but are expected to simulate real-world pooling conditions. The experiments in Figure 2 were performed by spiking NR-52350 directly onto a swab and then eluting that swab into NSB, PNM, or INM, which is now the commonly used method for determining LOD in data submitted to the FDA for EUA (Detect, 2021; USFDA, 2021). We alternatively used a pipettor to directly spike virus into PNM in the experiments depicted in Figures 3, 4, which allowed for more precise control over target concentration. The Accula’s stated LOD during the original EUA submission to the FDA was determined this way early during the pandemic.

To provide context for the interpretation of our measurements with the Accula system, one must consider the available options when screening a large group of people for SARS-CoV-2 infection. One strategy is to have all individuals at an event or gathering individually perform a test with home-use approval without the need for a certified laboratory space. This strategy is resource-intensive (one test is required for every participant) and the expected LOD is similar to those with home-use approval presented in Figure 5. A less resource-intensive strategy still employing home use tests would be to perform pooling with home-use tests. Our study does not evaluate LOD for pooling with tests other than the Accula system, but our observations with the Accula system suggest that compared to individual use, similar or worse performance (i.e., higher LOD) should be expected when pooling with any system.

An alternative approach is to perform pooling with a test that has been assigned a CLIA-waiver. This requires certification of a laboratory space by the Centers for Medicare and Medicaid Services (CMS) or approved accreditation agency, which may be appropriate for venues (such as school, offices, and event centers) with compatible facilities (CMS, 2021; Farmer et al., 2022). The data presented in Figure 5 demonstrate that many CLIA-waived tests without home-use approval claim superior LOD to home-use approved tests, although there are a few examples of tests with home-use approval with a comparable claimed LOD (Figure 5B). Ultimately, multiple factors such as cost and resource availability, assay time, and feasibility of obtaining a CLIA Certificate of Waiver in addition to diagnostic performance may influence the desired assay to be used in a pooling strategy. The comparison data shown in Figure 5 should also not be considered head-to-head comparisons of the likely performance of these assays in a swab pooling strategy, highlighting an opportunity for further research to better characterize additional technologies.

## 5. Conclusion

In summary, our data suggest that specimen pooling may have an inhibitory effect on LOD of the Accula SARS-CoV-2 RT-PCR assay and that this is likely due to a more significant burden of nasal mucous and debris in the pooled specimens. However, pooled PoC RT-PCR on the Accula platform still exhibits better LOD compared to individual use of most common FDA-approved home tests with a format compatible with swab pooling, and therefore warrants consideration in pooling strategies. Limitations of our work include the use of contrived specimens with heat inactivated virus and use of the United States-WA1/2020 isolate which is no longer representative of currently circulating variants, although assay performance of the Accula is not thought to be significantly affected by mutations in newer variants to date (USFDA, 2023). Further study is needed to validate our findings with clinical specimens and modern SARS-CoV-2 variants. Meanwhile, the impact of pooling should be investigated on other SARS-CoV-2 diagnostic test platforms and products, and real-world assessments should be done to clarify the costs and benefits of a pooling strategy compared to alternative approaches.

## Data availability statement

The original contributions presented in the study are included in the article/Supplementary material, further inquiries can be directed to the corresponding authors.

## Ethics statement

The studies involving humans were approved by Emory University Institutional Review Board. The studies were conducted in accordance with the local legislation and institutional requirements. The participants provided their written and/or verbal informed consent to participate in this study.

## Author contributions

ML, KF, and GD performed the Accula experiments. JS, WO, KM, HW, and JR supported the experiments and/or assisted with specimen collection, handling and storage. AR, LB, and MG designed and/or performed the antigen test LOD studies. PM, WK, ET, WL, ML, KF, and GD contributed to the design and interpretation of Accula experiments. All authors contributed to the article and approved the submitted version.

## Funding

This work was supported by NIH grant U54EB027690 to WL and NIH grant UL1TR002378.

## Conflict of interest

The authors declare that the research was conducted in the absence of any commercial or financial relationships that could be construed as a potential conflict of interest.

## Publisher’s note

All claims expressed in this article are solely those of the authors and do not necessarily represent those of their affiliated organizations, or those of the publisher, the editors and the reviewers. Any product that may be evaluated in this article, or claim that may be made by its manufacturer, is not guaranteed or endorsed by the publisher.

## Abbreviations

COVID-19: coronavirus disease 2019
SARS-CoV-2: Severe acute respiratory syndrome coronavirus 2
RT-PCR: reverse transcription polymerase chain reaction
PoC: point-of-care
FDA: United States Food and Drug Administration
CLIA: certificate of laboratory improvements amendments
qPCR: quantitative polymerase chain reaction
IFU: instructions for use
PNM: pooled nasal matrix
INM: individual nasal matrix
LOD: limit of detection
NSB: nasal swab buffer
EUA: emergency use authorization
MP: mechanical pipette
TP: transfer pipette
