# Supplementary material for: Effect of swab pooling on the Accula point-of-care RT-PCR for SARS-CoV-2 detection
Source: Front Microbiol. 2023 Aug 7;14:1219214. doi: 10.3389/fmicb.2023.1219214 (PMC10440424; doi:10.3389/fmicb.2023.1219214)
Supplement: Supplementary file 1 [file Data_Sheet_1.docx]

Supplementary Material

Effect of swab pooling on the Accula point-of-care RT-PCR for SARS-CoV-2 detection

Moira Lancelot^†,1^, Kirby Fibben^†,2^, Julie Sullivan^3^, William O’Sick^1^, Kaleb McLendon^1^, Huixia Wu^1^, Anuradha Rao^3,4^, Leda C. Bassit^3,4,5^, Morgan Greenleaf^3^, Pamela Miller^7^, Wolfgang Krull^7^, Erika Tyburski^3^, John D. Roback^1^, Wilbur A. Lam^*,2,3,4,8^, Gregory L. Damhorst^*,3,9^

*** Correspondence:** Corresponding Author: [gregory.damhorst@emory.edu](mailto:gregory.damhorst@emory.edu), [wilbur.lam@emory.edu](mailto:wilbur.lam@emory.edu)

# Supplementary Figures and Tables

**Supplementary Table 1.** LOD experiments with antigen tests from our institution using SARS-CoV-2 lineage B.1.2. These results were used to provide context for interpretation of the experiments presented here with the Accula platform. All experiments other than the Accula experiments were performed in matrix simulating specimen derived from a single individual (i.e. INM). INM, individual nasal matrix; PNM, pooled nasal matrix. *Indicates the experiments performed for pooling testing as presented in detail in this manuscript. Experiments for the other assays were performed as part of a separate testing program at our institution.

| **Manufacturer** | **SARS-CoV-2 test** | **Limit of detection**  **(copies per swab)** |
| --- | --- | --- |
| ANP Technologies, Inc. | NIDS COVID-19 Antigen Rapid Test Kit | 16,214 |
| GenBody Inc. | GenBody COVID-19 Ag | 81,143 |
| Xtrava Health | SPERA COVID-19 Ag Test | 21,071 |
| Quidel Corporation | QuickVue SARS Antigen Test | 116,500 |
| Quidel Corporation | Sofia 2 SARS Antigen FIA | 116,500 |
| Becton, Dickinson and Company (BD) | BD Veritor At-Home COVID-19 Test | 18,571 |
| Becton, Dickinson and Company (BD) | BD Veritor System for Rapid Detection of SARS-CoV-2 | 18,571 |
| Qorvo Biotechnologies, LLC. | Omnia SARS-CoV-2 Antigen Test | 6,829 |
| Thermo Fisher Scientific Inc. | Accula SARS-Cov-2 Test, INM* | 2,250* |
| Thermo Fisher Scientific Inc. | Accula SARS-Cov-2 Test, PNM* | 3,750* |


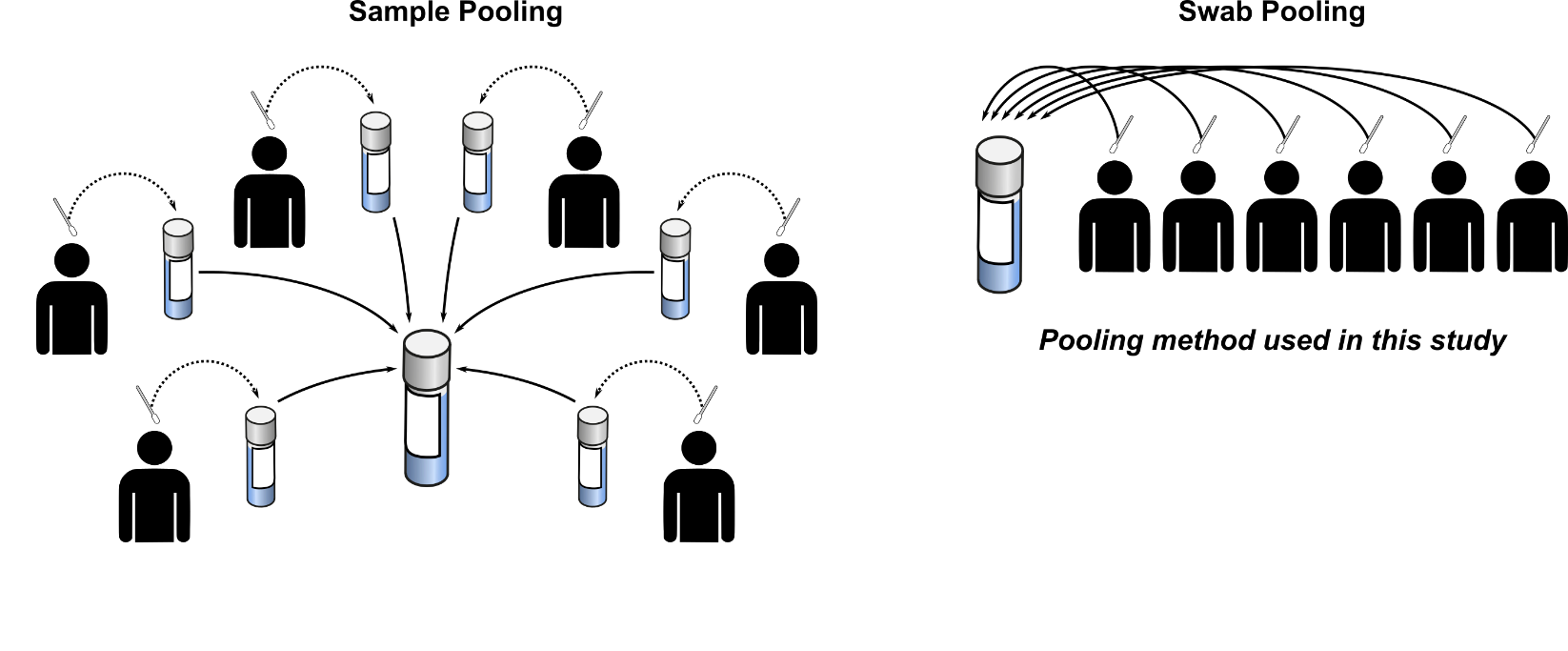


**Supplementary Figure 1.** Schematic of sample pooling (left) versus swab pooling (right).

**Supplementary Figure 2.** Standard curve used for quantification of viral RNA in LOD comparison experiments presented in Figure 5. Cp = crossing point.
